# Comprehensive Multi‐Omics Analysis Reveals NPC2 and ITGAV Genes as Potential Prognostic Biomarkers in Gastrointestinal Cancers

**DOI:** 10.1002/cnr2.70087

**Published:** 2024-12-17

**Authors:** Moein Piroozkhah, Mohammadreza Zabihi, Pooya Jalali, Zahra Salehi

**Affiliations:** ^1^ Basic and Molecular Epidemiology of Gastrointestinal Disorders Research Centre, Research Institute for Gastroenterology and Liver Diseases Shahid Beheshti University of Medical Sciences Tehran Iran; ^2^ Institute of Biochemistry and Biophysics (IBB), Department of Bioinformatics, Laboratory of Complex Biological Systems and Bioinformatics (CBB) University of Tehran Tehran Iran; ^3^ Hematology, Oncology and Stem Cell Transplantation Research Center, Research Institute for Oncology, Hematology and Cell Therapy Tehran University of Medical Sciences Tehran Iran

**Keywords:** bioinformatics, gastrointestinal cancer, ITGAV, multiomics, NPC2, prognostic factors

## Abstract

**Background:**

Gastrointestinal cancers (GICs) continue to dominate in terms of both incidence and mortality worldwide. Due to the absence of efficient and accurate prognostic biomarkers, the prognosis and treatment outcomes of many GICs are poor. Identifying biomarkers to predict individual clinical outcomes efficiently is a fundamental challenge in clinical oncology. Although several biomarkers have been continually discovered, their predictive accuracy is relatively modest, and their therapeutic use is restricted. In light of this, the discovery of reliable biomarkers for predicting prognosis and outcome in GIC is urgently required.

**Materials and Methods:**

We evaluated the Human Protein Atlas dataset and identified NPC Intracellular Cholesterol Transporter 2 (*NPC2*) and Integrin Subunit Alpha V (*ITGAV*) as probable poor predictive genes for these cancers. In addition, we used the GEPIA2, cBioPortal, UALCAN, LinkedOmics, STRING, Enrichr, TISDB, TIMER2.0, hTFTarget, miRTarBase, circBank, and drug–gene interaction database databases to conduct a comprehensive and systematic analysis of the *NPC2* and *ITGAV* genes.

**Result:**

Our results found high expression levels of *NPC2* and *ITGAV* in most GICs. The aforementioned gene expressions were linked to several clinicopathological characteristics of GICs as well as poorer prognosis in LIHC and STAD. The most common alteration type of *NPC2* was amplification, and for *ITGAV* was deep deletion. Significant promotor hypermethylation was also seen in *NPC2* and *ITGAV* in PAAD and COAD, respectively. For the immunologic significance, *NPC2* and *ITGAV* were positively correlated with the abundance of tumor‐infiltrating lymphocytes and macrophages. Furthermore, various immunomodulators showed strong correlations with the expression of these genes. There were currently 10 small molecule drugs targeting *ITGAV*.

**Conclusion:**

Consequently, our bioinformatics analysis showed that *NPC2* and *ITGAV* might be used as potential biomarkers to determine the prognosis of various GICs and are also related to immune infiltration.

**Abbreviations**
BP: biological process
CC: cellular component
ceRNA: competing endogenous RNA
CNV: copy number variation
COAD: colon adenocarcinoma
DFS: disease‐free survival
EMT: epithelial‐mesenchymal transition
ESCA: esophageal carcinoma
GI: gastrointestinal
GICs: gastrointestinal cancers
GO: Gene Ontology
GRN: gene regulatory networks
HPA: Human Protein Atlas
ICIs: immune checkpoint inhibitors
IHC: immunohistochemistry
ITGAV: Integrin Subunit Alpha V
KEGG: Kyoto Encyclopedia of Genes and Genomes
LIHC: liver hepatocellular carcinoma
MF: molecular function
NK: natural killer cells
NPC2: NPC Intracellular Cholesterol Transporter
OS: overall survival
PAAD: pancreatic adenocarcinoma
PPI: protein–protein interaction
PTM: posttranslational modification
READ: rectum adenocarcinoma
STAD: stomach adenocarcinoma
TF: transcription factor
TGF: tumor growth factor
TIME: tumor immune microenvironment
TTR: time to recurrence
YY1: Yin Yang 1

## 1 Introduction

With an estimated 5 million new cases and 3.5 million deaths worldwide in 2020, gastrointestinal cancers (GICs) represent over one‐quarter (26%) of the global cancer incidence and over one‐third (35.4%) of all cancer‐related deaths [1]; to such an extent that six GICs, including liver, stomach, colon, esophagus, pancreas, and rectum cancers, are in the top 10 for death rates from all tumors [1]. Histopathological parameters of tumor invasion, according to the American Joint Committee on Cancer and Union for International Cancer Control TNM classification system, are used to predict the prognosis of patients with GICs [2, 3]. Nevertheless, several studies have shown that, even among patients with the same TNM stage [4], there may be significant variation in their clinical outcomes despite receiving the same treatment, owing to the genetic and epigenetic heterogeneity of cancer [5, 6]. Given this, it is essential to comprehend the molecular mechanisms underlying cancer to develop efficient prognostic and predictive biomarkers for clinical research and practice [7]. Only a small number of the cancer biomarkers discovered in recent decades are presently employed in clinical practice, indicating that there is still room to enhance biomarkers and open the door to more personalized therapy approaches [8, 9].

Nowadays, promising cancer prevention and screening particularly show that understanding pan‐cancer development would provide novel opportunities for confronting diverse malignant tumors [10]. As a result, identifying the hub tumor‐related genes is critical for developing efficient diagnostic and prognostic biomarkers as well as therapeutic targets. Massive high‐throughput data and various publicly accessible big data online public databases are advantageous for identifying tumorigenic genes and undertaking pan‐cancer investigations [11, 12, 13]. In that context, the focus of a body of research has been the discovery of efficacious comprehensive markers for predicting the prognosis and treatment for GI cancer to a better prognosis and fewer side effects. As shown by Fang et al. [14] *SNAI1* can be used as a prognostic biomarker for determining prognosis and immune infiltration in GICs. Pan et al. [15] comprehensively analyzed *LAYN* expression and its relationship to prognosis in numerous cancers across several databases, concluding that *LAYN* may be employed as a predictive biomarker for prognosis and immune infiltration in colon and gastric cancer.

To the best of our knowledge, an in‐depth analysis to explore biomarkers for the prognosis of GIC patients, in light of the most up‐to‐date knowledge, remains largely unknown. Herein, we use bioinformatics databases to comprehensively and systematically discover the role of NPC Intracellular Cholesterol Transporter (*NPC2*) and Integrin Subunit Alpha V (*ITGAV*) as potential unfavorable prognostic biomarkers for GICs. The result indicated that the expression levels of *NPC2* and *ITGAV* are related to GICs prognosis and tumor—immune signature. Additionally, it was looked into whether the mentioned genes might have a role in the pathophysiology of GICs. Finally, we explored the *NPC2* and *ITGAV* regulatory networks. In summary, these results may give a new avenue for the development of useful prognostic biomarkers for GICc patients.

## 2 Materials and Methods

**FIGURE 1:**
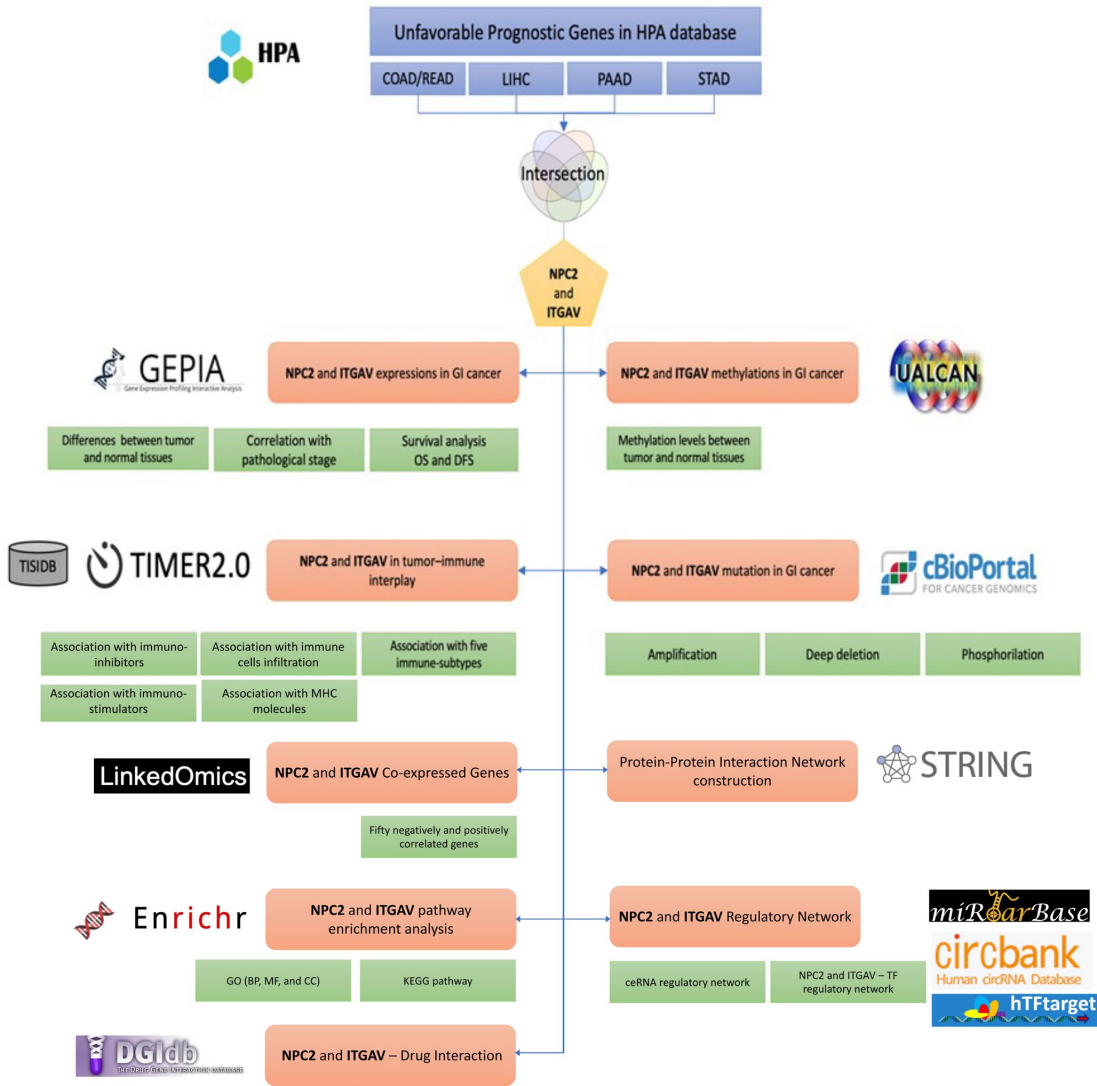
A schematic illustration of the bioinformatics analysis in our study. At first, the HPA database was mined for all of the unfavorable genes associated with COAD/READ, LIHC, PAAD, and STAD. The overlap between these genes in four different datasets was represented using a Venn diagram. Consequently, *NPC2* and *ITGAV* were chosen to serve as potential predictive biomarkers. We then comprehensively and systematically analyzed the *NPC2* and *ITGAV* genes using the GEPIA2 for expression analysis, cBioPortal for genetic alteration, UALCAN for methylation analysis, TISIDB and TIMER2.0 databases for immune signature analysis, LinkedOmics for co‐expression genes, STRING for PPI construction, Enrichr for enrichment pathway analysis, miRTarBase and CircBank for ceRNA network prediction, hTFTarget for TF–genes network construction, and DGIdb database for drug–gene interaction. PPI: protein–protein interaction; ceRNA: complementary endogenous RNA; TF: transcription factor.

Through the Human Protein Atlas (HPA) database, we first extracted all the unfavorable prognostic genes of the COAD/READ, LIHC, PAAD, and STAD. By the intersection of these four datasets, we concluded that NPC2 and ITGAV (integrin alpha V subunit) are common among most of these cancers. Then via several bioinformatics databases, we comprehensively and systematically explored the roles of these two genes in the most prevalent GICs. Employing the GEPIA2, UALCAN, and cBioPortal databases, we analyzed the expression, methylation, and genetic alterations of *NPC2* and *ITGAV*. Further, co‐expression genes, protein–protein interaction (PPI) networks, and pathway enrichment analysis of mentioned genes were scrutinized by the LinkedOmics, STRING, and Enrichr databases, respectively. Then, we explored the correlation of *NPC2* and *ITGAV* with tumor‐immune signatures in the different tumor microenvironments via TISIDB and TIMER2.0. Predicting *NPC2* and *ITGAV* regulatory networks, including competing endogenous RNA (ceRNA) and TF—genes network, was another part of our investigation. Eventually, we inspected the drug–gene interaction database (DGIdb) database for *NPC2* and *ITGAV* interacted drugs. The schematic analysis steps are illustrated in Figure 1.

### 2.1 HPA Database

The HPA (https://www.proteinatlas.org) [16] is an online database for exploring the human proteins using omics data integration in 17 major cancer types, additionally offering diagnostic and prognostic data on the protein expression profiles of a variety of cancers, normal tissues, and cell lines based on immunohistochemistry. Here, we combed through databases of prognostic proteins in four gastrointestinal malignancies (PAAD, STAD, LIHC, and COAD/READ) to identify unfavorable prognostic proteins that were common to all of them. A Venn diagram tool (https://bioinformatics.psb.ugent.be/webtools/Venn/) was employed to display the intersection of these four datasets.

### 2.2 cBioPortal Database

cBioPortal (https://www.cbioportal.org) [17] is an open‐source platform for visualizing and analyzing multidimensional cancer genomes and clinical data. The public cBioPortal offers over 200 cancer genome studies, including all TCGA data and many other datasets selected from the literature. Its biologist‐friendly interface includes a graphical overview of gene‐level data across several platforms, correlation analysis between genes or other data types, survival analysis, and per‐patient data visualization. The cBioPortal was used to examine and visualize *NPC2* and *ITGAV* genetic alteration in four GIC cohorts, including structural variation data, copy number variation, and mutation.

### 2.3 UALCAN Database

The UALCAN database (http://ualcan.path.uab.edu/index.html) allows genomics, bioinformatics, and integrative approach to better understand the molecular mechanism of cancer [18]. The UALCAN database was utilized in this work to evaluate the methylation and protein phosphorylation levels of the *NPC2* and *ITGAV* promoter regions in normal and cancer tissues. The significance of differences was determined using a student's *t* test, in which *p* value < 0.05 was considered significant.

### 2.4 GEPIA2 Database

GEPIA2 (http://gepia2.cancer‐pku.cn/#index) is a comprehensive cancer database based on TCGA and GTEx data that contains gene expression and prognostic information for over 30 prevalent human malignancies [19, 20]. In this investigation, we utilized the GEPIA2 Database to analyze the expression of *NPC2* and *ITGAV* in digestive tract tumor tissues and their matched normal tissues and normal individuals. We exhibited the results using BodyMap, dot plot, and box plot. Following that, we utilized this Database to investigate the relationship between *NPC2* and *ITGAV* expression and tumor pathological stage. All of the above use log2 (TPM+1) as the log scale. Besides that, we utilized the “Survival Plots” module to investigate the link between *NPC2* and *ITGAV* expression and four GIC prognosis.

### 2.5 LinkedOmics Database

LinkedOmics (http://linkedomics.org/admin.php) is an online portal with multi‐omics data from 32 TCGA Cancer types [21]. The LinkedOmics website allowed a flexible exploration of associations between a molecular or clinical attribute of interest and all other attributes, providing the opportunity to analyze and visualize associations between billions of attribute pairs for each cancer cohort. This study utilized LinkedOmics to examine *NPC2* and *ITGAV* co‐expression models from TCGA GI cohorts. *NPC2* and *ITGAV* co‐expression was analyzed statistically using Pearson's correlation coefficient and finally presented in volcano plots and heat maps.

### 2.6 STRING Database

PPI data can be analyzed using the STRING database (https://cn.string‐db.org) [22]. This Database was used in our study to investigate the PPI network of *NPC2* and *ITGAV*. To achieve this, we independently selected the protein names “*NPC2*” and “*ITGAV*” and the organism “ *Homo sapiens* ” from the STRING website. Then, we adjusted the primary settings such that we have a minimum interaction level of “low confidence [0.150]” maximum number of displayed interaction factors (“no more than 50 interactors in the first shell”), the meaning of the network edge (“evidence”), and active interaction sources (“experiments”). Furthermore, we obtained experimentally determined proteins that bind NPC2 and ITGAV. Our study's false discovery rate (FDR) was less than 0.01.

### 2.7 Enrichr Database

The Enrichr database (http://amp.pharm.mssm.edu/Enrichr/) database is a comprehensive online server for gene set enrichment analysis [23, 24]. Gene Ontology (GO) and Kyoto Encyclopedia of Genes and Genomes (KEGG) pathway enrichment analysis for *NPC2* and *ITGAV* genes was performed using the Enrichr dataset in the present investigation. GO terms consist of biological process (BP), cellular component (CC), and molecular function (MF).

### 2.8 TISIDB Database

TISIDB (http://cis.hku.hk/TISIDB/index.php) is a web service for assessing the interrelations between cancers and the immune system. It integrates multiple diverse data types, such as literature mining results from the PubMed Database, high‐throughput screening data, exome, and RNA sequencing datasets of patient cohorts with immunotherapy, genomics, transcriptomics, and clinical information of 30 cancer types from TCGA and public databases [25]. We investigated the association between *NPC2* and *ITGAV* expression and tumor immune‐related components using this information. In addition, the TISIDB database was used to examine the expression levels of *NPC2* and *ITGAV* in various immunosubtype of selected cancers.

### 2.9 TIMER2.0 Database

TIMER2.0 (http://timer.cistrome.org) is an immune evaluation tool based on the expression of tumor gene signatures that incorporates 10 897 samples from TCGA for a comprehensive analysis of immune infiltrates cells information across 32 distinct cancer types [26]. In this study, we examined the relationship between *NPC2* and *ITGAV* expression and the levels of six types of immune cells, including CD8+ T cells, CD4+ T cells, B cells, macrophages, neutrophils, and dendritic cells.

### 2.10 hTFtarget Database

Bioinformatics prediction website hTFtarget (http://bioinfo.life.hust.edu.cn) has compiled detailed transcription factor (TF)—target regulations from massive ChIP‐Seq data of human TFs (7190 experiment samples of 659 TFs) across 569 conditions (399 cell‐line types, 129 classes of tissues or cells, and 141 types of treatments) [27]. Here, we selected only the TFs that target *NPC2* and *ITGAV* in the tissues of the GI tract. Using the Venn diagram (https://bioinformatics.psb.ugent.be/webtools/Venn/), the overlapping TFs of *NPC2* and *ITGAV* in hTFtargets were identified. The TF‐Genes regulatory network was built and mapped utilizing the Cytoscape tool (version 3.9.1).

### 2.11 miRTarBase Database

The miRTarBase Database (http://mirtarbase.mbc.nctu.edu.tw) already contains over 360 thousand miRNA–target interactions that have been experimentally validated [28]. The miRTarbase Database was utilized in this investigation to anticipate miRNA interactions with the NPC2 and ITGAV mRNAs.

### 2.12 CircBank Database

A large collection of human circRNA with predicted binding miRNA can be found in the circBank Database (http://www.circbank.cn/) [29]. The circBank database stores circRNAs and is accessible to the public for searching, browsing, and downloading. The Database may be utilized as a tool to facilitate research into the function and regulation of circRNAs. Using this Database, we determined the potential interactions between the selected miRNAs and circRNAs.

### 2.13 DGIdb Database

The DGIdb (http://www.dgidb.org/) database is an online resource that compiles drug–gene interactions from various sources, including databases and text mining [30]. This study utilized the DGIdb database to identify the drugs that interact with *NPC2* and *ITGAV*. Those found in DGIdb were compared to those that have been or are being studied for the treatment of CRC in the ClinicalTrials.gov database [31].

## 3 Results

### 3.1 NPC2 And ITGAV Shared the Maximum Overlap Across the GIC Types

**FIGURE 2:**
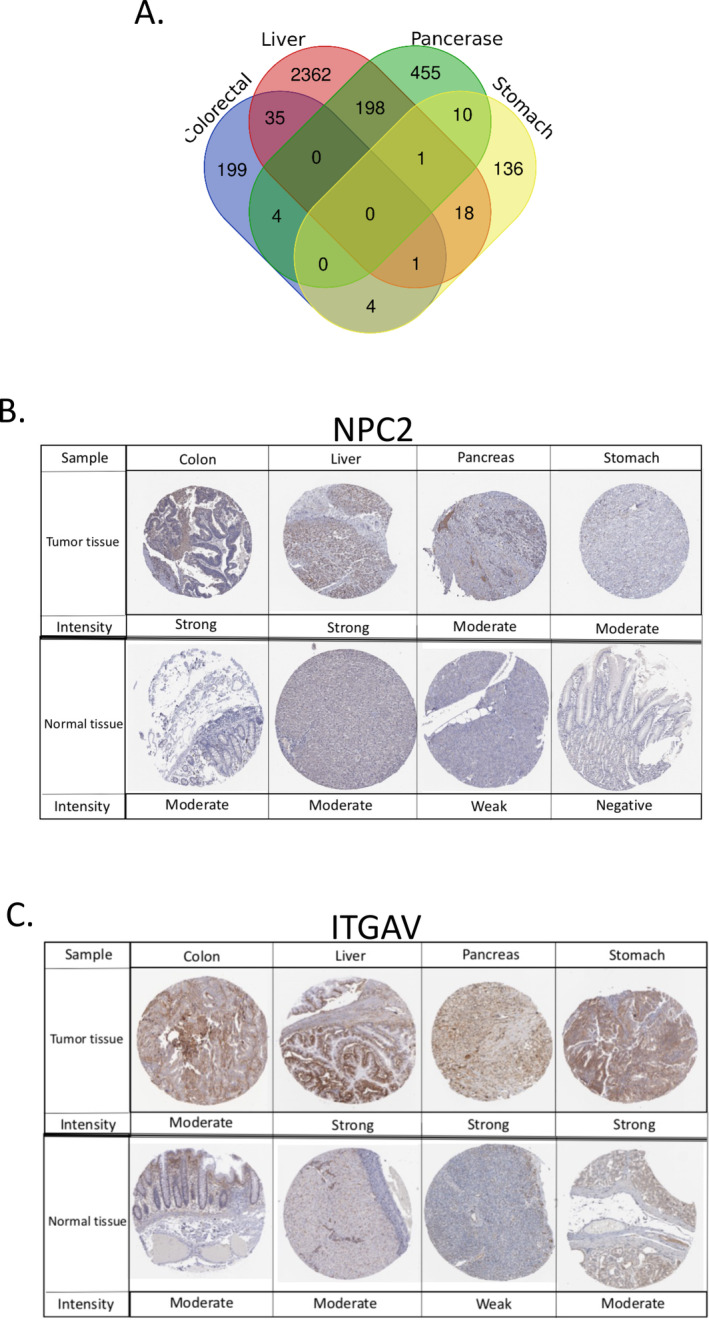
Identification of most common genes with unfavorable prognostic values in GICs and their protein expression in GI cancers and tissues (a) Four‐set Venn diagram shows genes with maximum overlap from PAAD, LIHC, STAD, and COAD/READ unfavorable gene series based on HPA datasets. (B, C) Comparison of *NPC2* (B) and *ITGAV* (C) protein expressions between normal and tumor tissues shown by immunohistochemistry images based on the HPA database. GICs: gastrointestinal cancer, HPA: Human Protein Atlas; *NPC2*: NPC Intracellular Cholesterol Transporter 2; *ITGAV*: Integrin Subunit Alpha V.

The search for prognostic cancer genes with unfavorable values was sought to utilize the HPA database. For PAAD, LIHC, STAD, and COAD/READ, the HPA database was mined to retrieve 668, 2615, 179, and 243 genes with poor prognosis, respectively (Table S1). Venn analysis was carried out to investigate the intersection between the unfavorable genes across these cancers (Table S2). The list was narrowed down to the two candidate genes, which shared the maximum overlap between the cancer types, including *NPC2* and *ITGAV* (Figure 2A). It was shown that *NPC2* was common in COAD/READ, LIHC, and STAD, whereas *ITGAV* was prevalent in PAAD, LIHC, and STAD. In addition, to further verify the protein level of *NPC2* and *ITGAV* expression, we compared the IHC results from the HPA database; and we found that the *NPC2* and *ITGAV* proteins were expressed higher in tumor tissues than the normal ones (Figure 2B,C).

*NPC2* is a protein‐coding gene with a lipid recognition domain that has been linked to lipoprotein metabolism and the innate immune pathway. *NPC2* binds free cholesterol and modulates intracellular free cholesterol transportation and homeostasis [32]. *ITGAV* is a Protein Coding gene. This gene's product is a member of the integrin alpha chain family. Integrins are heterodimeric integral membrane proteins consisting of alpha and beta subunits that act in adhesion and signaling at the cell surface. Hydrolysis of the coding proprotein yields light and heavy chains containing the alpha V subunit. *ITGAV* consists of five members αvβ1, αvβ3, αvβ5, αvβ6, and αvβ8 and is a component of the receptors for fibronectin, vitronectin, and fibrinogen [33].

### 3.2 The mRNA Expression Landscape of NPC2 and ITGAV in GICs

**FIGURE 3:**
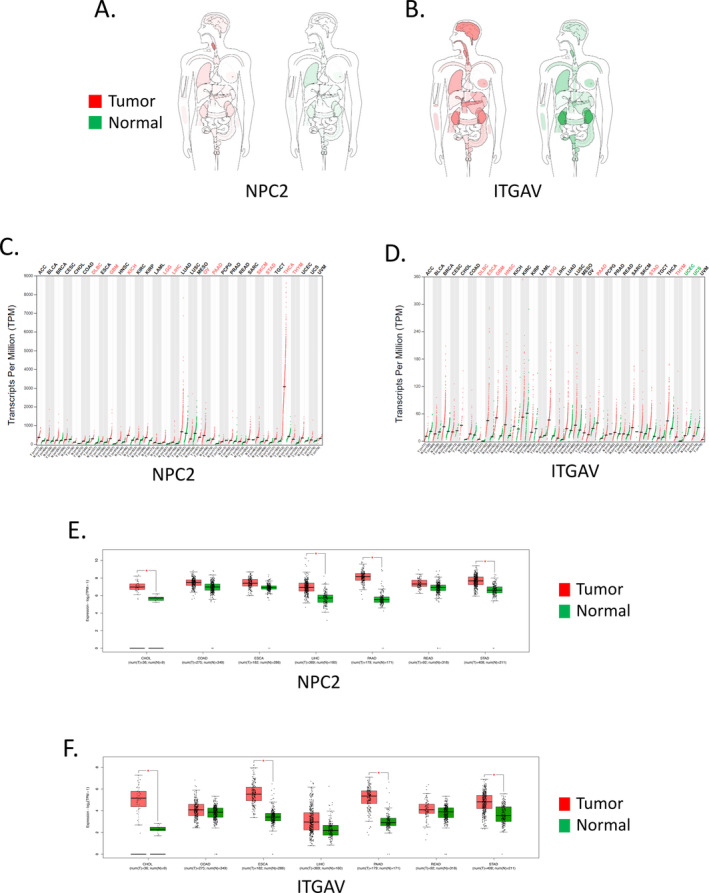
The mRNA expression landscape of *NPC2* and *ITGAV* in human pan‐cancer based on the GEPIA2 database. (A, B) Interactive BodyMaps; (C, D) dot plot; and (E, F) box plot. Each dot represents the expression of samples.

We comprehensively investigated *NPC2* and *ITGAV* mRNA expression levels using the GEPIA2 dataset. Based on the overall level of the interactive BodyMap, we discovered that the expression levels of *NPC2* and *ITGAV* in most human cancers and comparable normal tissues varied, notably in the digestive organs (esophagus, pancreas, liver, and stomach), as well as the brain, blood, thyroid, and other organs and tissues (Figure 3A,B). Following this, we investigated the mRNA expression levels of *NPC2* and *ITGAV* in 33 human cancers and matched normal tissues and healthy individuals (Figure 3C,D). Among GICs, *NPC2* mRNA expression was significantly higher in LIHC, PAAD, and STAD (Figure 3E); *ITGAV* mRNA expression was considerably higher than the average level in ESCA, PAAD, and STAD compared to normal tissues (Figure 3F).

### 3.3 Correlations Between the NPC2 and ITGAV Expression and Tumor Pathological Stage

**FIGURE 4:**
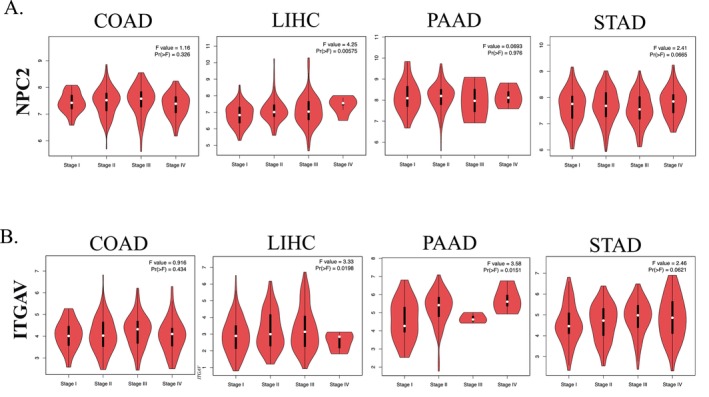
Correlations between the genes' expression and GICs pathological stage based on GEPIA2. (A) Association between *NPC2* mRNA expression and COAD, LIHC, PAAD, and STAD's pathological stage. (B) Association between *ITGAV* mRNA expression and COAD, LIHC, PAAD, and STAD's pathological stage. Log2 (TPM+1) was used for the log scale. TPM: Transcripts Per Million.

The pathological stage of the tumor is a significant indication of the patient's prognosis. Consequently, we revisited the GEPIA2 dataset to investigate if there was a linkage between *NPC2* and *ITGAV* expression and the pathogenic stage of GICs. Our findings indicated that, surprisingly, *NPC2* expression was substantially associated with tumor stage in LIHC (*p* value = 0.00575) (Figure 4A). ITGAV expression was strongly correlated with tumor stage in LIHC (*p* value = 0.0198) and PAAD (*p* value = 0.0151) (Figure 4B). Other tumor pathological stages were not associated with these gene expression levels (*p* value > 0.05) (Figure 4). Collectively, our findings show that *NPC2* and *ITGAV* expression levels are linked to the pathological staging of LIHC and PAAD, which may serve as a valuable guide for clinical stage assignment.

### 3.4 The Relationship Between NPC2 and ITGAV Expression Levels and Prognosis of Tumor Patients

**FIGURE 5:**
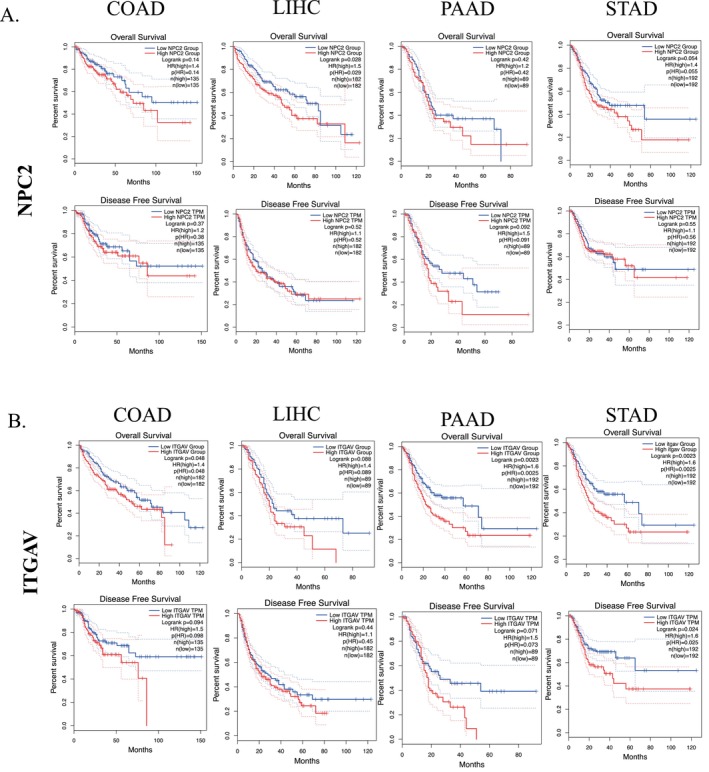
KM survival curves of the prognostic significance of high‐ and low‐genes expression based on GEPIA2. (A, B) Association between *NPC2* (A) and *ITGAV* (B) expressions with OS and DFS of patients with different types of GICs with a 95% CI. The first and third panels show the survival curves for OS, and the second and fourth panels show the survival curves for DFS. KM: Kaplan–Meier, OS: overall survival, DFS: disease‐free survival, GIC: gastrointestinal cancer, CI: confidence interval.

To explore the prognostic value of the *NPC2* and *ITGAV* in patients with GI cancer, we assessed the impact of differentially expressed *NPC2* and *ITGAV* on clinical outcomes, including overall survival (OS) and disease‐free survival (DFS) using GEPIA2. The Kaplan–Meier survival curves of the results from four types of GIC suggest that high *NPC2* expression was significantly associated with worse OS in LIHC (log‐rank *p* = 0.028) (Figure 5A). In addition, we also found that high expression of *ITGAV* in LIHC (OS: log‐rank *p* = 0.048) and STAD (OS: log‐rank *p* = 0.0023; DFS: log‐rank *p* = 0.024) have a worse prognosis (Figure 5B) and there was no statistical difference between the high or low expression of *NPC2* and *ITGAV* in other tumors' OS and DFS.

### 3.5 NPC2 and ITGAV Genetic Alteration Analysis

**FIGURE 6:**
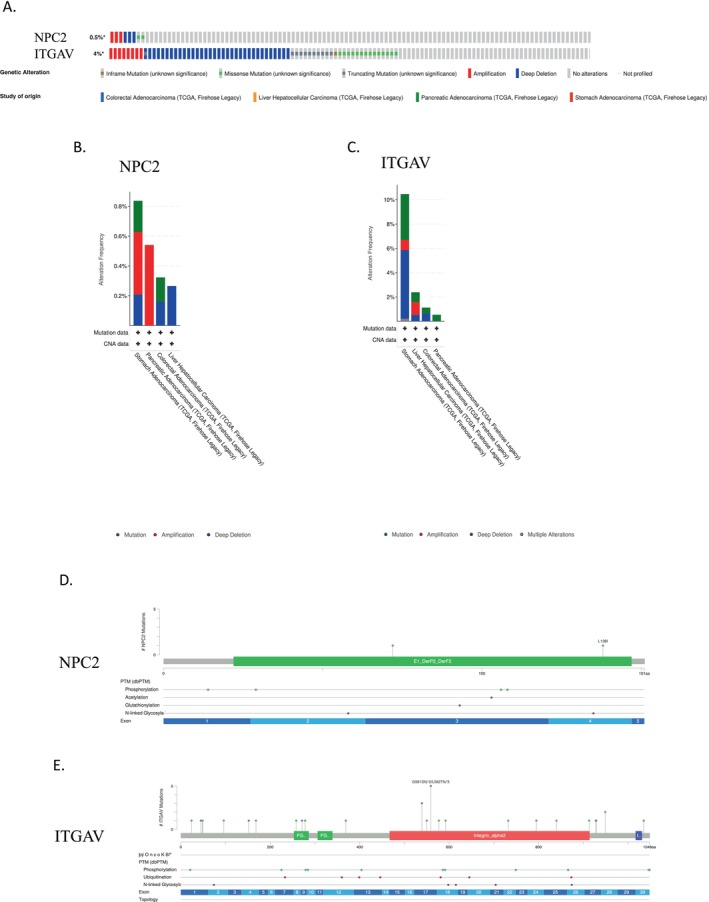
Analysis of *NPC2* and *ITGAV* gene mutation and level in GICs. (A) The total alterations in the *NPC2* and *ITGAV* genes were assessed using a genome‐wide GI‐cancer analysis in the cBioPortal database (TCGA, Firehose Legacy). (B, C) The *NPC2* (B) and *ITGAV* (C) gene alteration frequency with different types of mutations. (D, E) Mutation diagram and PTM data of *NPC2* (D) and *ITGAV* (E) in different digestive cancer types across protein domains. PTM: posttranslational modification.

Genetic mutations significantly influence cancer development, and these altered genes may potentially serve as useful prognostic biomarkers [34]. To dissect the molecular landscape of *NPC2* and *ITGAV* alterations in GICs, at the first step, we conducted an in‐depth study using the cBioPortal Database and found that *NPC2* and *ITGAV* were altered in 0.5% (8/1659) and 4% (67/1659) of patients in four publicly available datasets, respectively (Figure 6A). Furthermore, we also analyzed the alteration frequency of the *NPC2* and *ITGAV* genes in these types of tumors. As Figure 6B,C shows, amplification is the most frequent form of *NPC2* gene mutation, while deep deletion is the most common form of ITGAV gene mutation. Intending to decipher the mutational landscape of *NPC2* and *ITGAV* in digestive cancer types across protein domains, we conducted an in‐depth analysis, which revealed a total of two and 28 mutation sites, respectively (Figure 6D,E). According to the posttranslational modification (PTM) data in Figure 6C,D, Phosphorylation modification was the most common kind of PTM in *NPC2* and *ITGAV*, with four and 11 sites, in order.

### 3.6 Analysis of NPC2 and ITGAV Methylation Level in GICs

**FIGURE 7:**
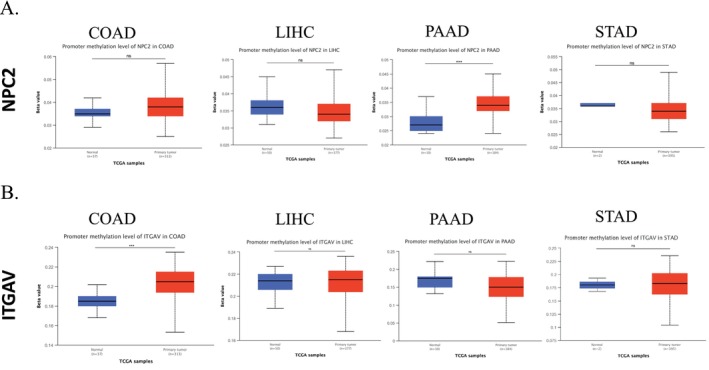
Analysis of *NPC2* and *ITGAV* methylation level in GIC. Analysis of *NPC2* (A) and *ITGAV* (B) methylation levels in COAD, LIHC, PAAD, and STAD based on the UALCAN database. (ns: *p* value > 0.05 and *: *p* value < 0.05). ns: not significant.

One other significant factor in the development of cancer is aberrant DNA methylation [31]. For this reason, we next investigated *NPC2* and *ITGAV* methylation in GICs and corresponding tissues using the UALCAN database (Figure 7A,B). Our results indicate that, compared with normal tissues, *NPC2* methylation levels were significantly increased in PAAD tissues (Figure 7A), and *ITGAV* methylation was increased considerably in COAD tissues (Figure 7B).

### 3.7 Gene Co‐Expression, Protein–Protein Networks Construction, and Pathway Enrichment Analysis

**FIGURE 8:**
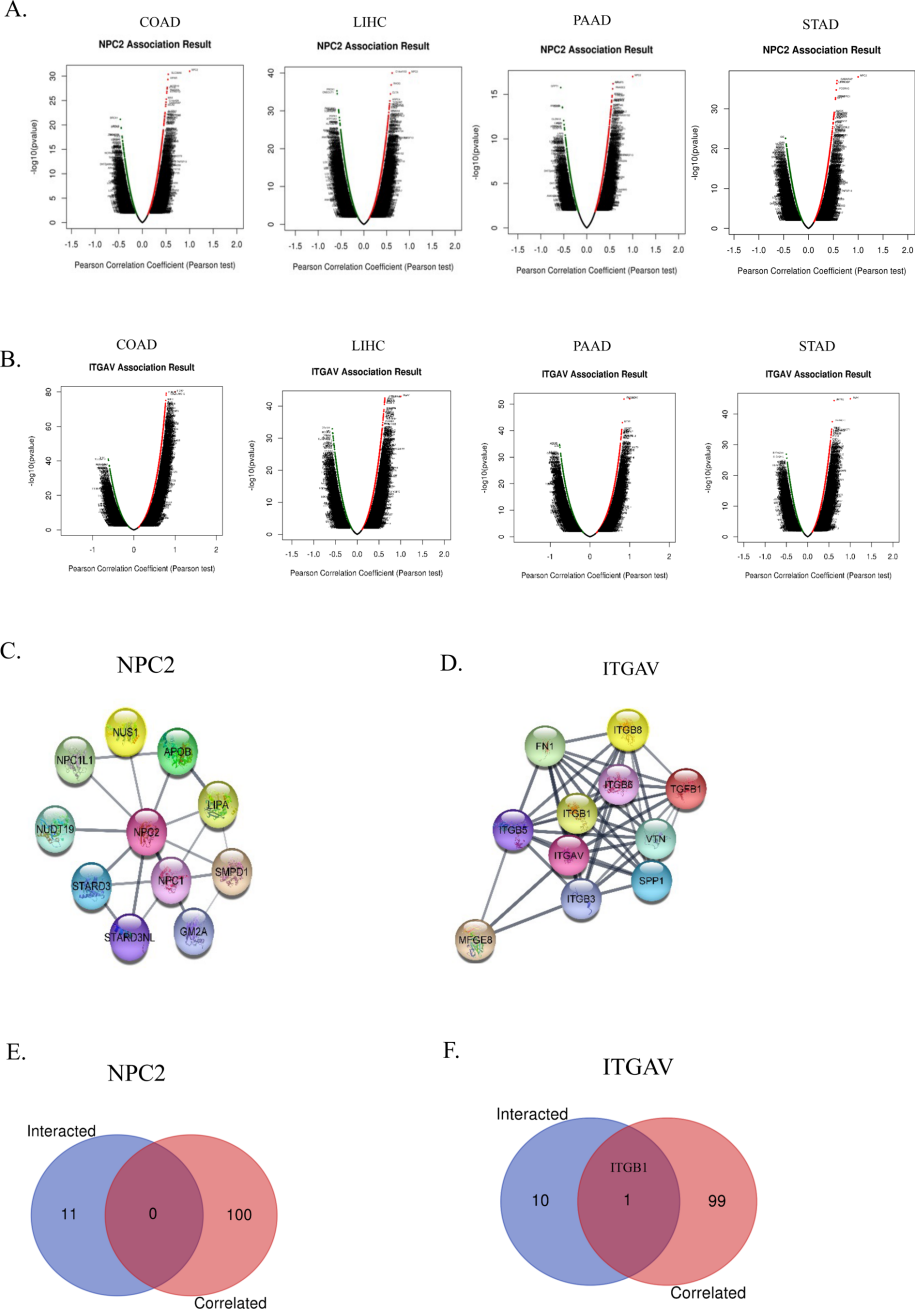
Co‐expression genes of *NPC2* and *ITGAV* in GICs (LinkedOmics) and protein–protein interaction (PPI) network construction (STRING). (A, B) Volcano Plot demonstrating the positively and negatively correlated genes with *NPC2* (A) and *ITGAV* (B). (C, D) Predicting the PPI network of *NPC2* (C) and *ITGAV* (D) using STRING. (E, F) Intersection analysis of *NPC2* (E) and *ITGAV* (F) correlated and interacted proteins.

We postulated that *NPC2* and *ITGAV*'s functions in GICs could be intimately tied to that of its adjacent genes. In light of this, the co‐expression genes were analyzed in the TCGA GI cohort using the LinkedOmics database. Genes positively or negatively correlated with *NPC2* (Figure 8A) and *ITGAV* (Figure 8B) were represented by dark red dots and dark green pots in volcano plots. Supplementary Figure S1A,B also demonstrated heat maps of the positive and negative correlations between *NPC2* and *ITGAV* with 50 significant gene sets, respectively.

Using the STRING website, we constructed a PPI network to assess the interactions for NPC2 and ITGAV better. The PPI networks include 11 nodes and 19 edges with an average node degree of 3.45 For *NPC2* (Figure 8C), and 11 nodes and 48 edges with an average node degree of 8.73 for *ITGAV* (Figure 8D), respectively. Furthermore, we compared 50 interacted proteins to the top 100 correlated genes with *NPC2* and *ITGAV* derived from the GEPIA2 Database, which revealed no common gene for NPC2 (Figure 8E) and ITGB1 for ITGAV (Figure 8F).

Subsequently, GO and KEGG pathway enrichment analyses of *NPC2* and *ITGAV* were performed by combining their 100 correlated genes and binding proteins. According to the findings, the most significant BP, MF, CC, and KEGG pathway analyses for *NPC2* were sterol transport (GO:0015918), cholesterol binding (GO:0015485), and lysosomal lumen (GO:0043202), respectively (Figure S1C). Also, the most significant BP, MF, and cellular function for *ITGAV* were cell‐matrix adhesion (GO:0007160), C‐X3‐C chemokine binding (GO:0019960), and focal adhesion (GO:0005925), respectively. The most significant KEGG pathway analyses for *ITGAV* were the regulation of the actin cytoskeleton (Figure S1D).

### 3.8 Association Between NPC2 and ITGAV With Immune‐Related Signatures

**FIGURE 9:**
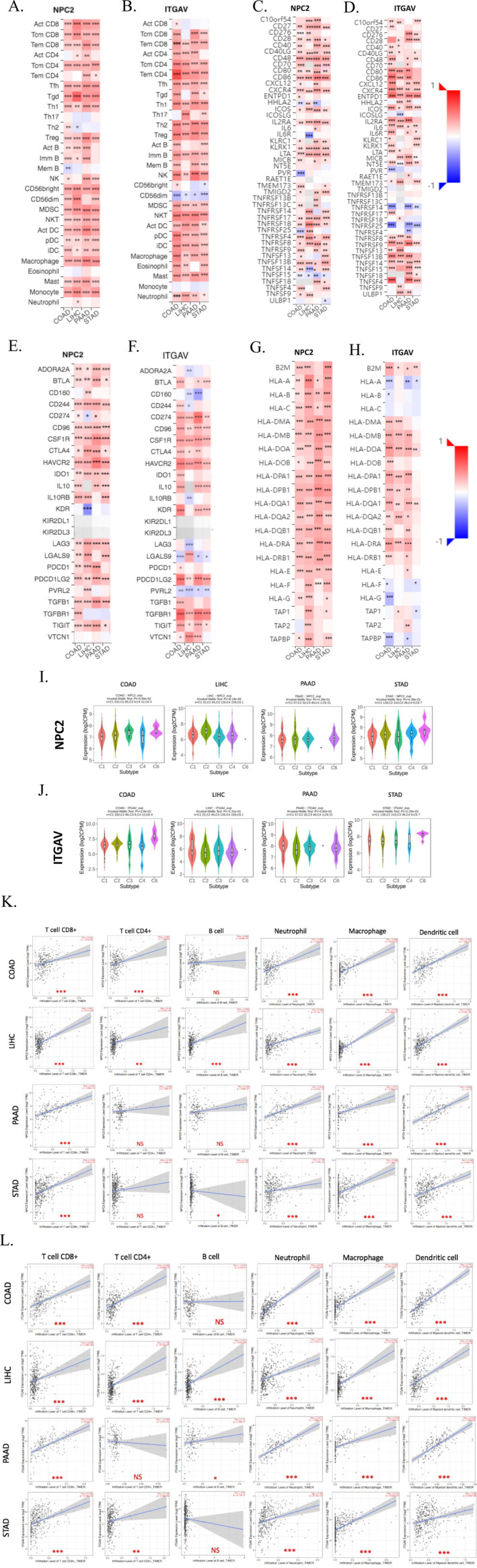
The relationship between *NPC2* and *ITGAV* gene expression with immune‐related signatures in GICs. (A–H) Heat‐map of analysis between *NPC2* and *ITGAV* genes expression with immune infiltration levels (A, B); immune‐stimulators level (C, D); immuno‐inhibitors levels (E, F); and MHC levels (G, H). (I, J) Association between five immune subtypes with *NPC2* (I) and *ITGAV* (J) expression based on the TISIDB database. (K, L) Correlation between *NPC2* (K) and *ITGAV* (L) gene expression related to immune infiltration of CD8+ T cells, CD4+ T cells, B cells, Neutrophils, and macrophage cells Dendritic cells in GICs by TIMER02. (NS: *p* value > 0.05, *: *p* value < 0.05, **: *p* value < 0.01, ***: *p* value < 0.001). MHC: major histocompatibility complex, NS: nonsignificant.

It is well‐established that tumor‐infiltrating immune cells play a crucial role in tumor development and progression. In addition, immunomodulators and cytokines in the tumor‐immune microenvironment (TIME) may significantly impact infiltrating immune cells' activity [35, 36]. There is also evidence that immune cells may serve as an independent predictor of survival and chemotherapy response [37, 38]. Therefore, evaluating the relationship between *NPC2* and *ITGAV* with TIME is necessary. We initially performed a GIC analysis of the *NPC2* and *ITGAV* gene expression with infiltration of immune cells using the TISIDB database (Figure 9A,B, respectively). *NPC2* expression was the most closely related to infiltrating levels of gamma delta T cell in COAD (*r* = 0.33, *p* = 5.30e‐13) and macrophage in PAAD (*r* = 0.452, *p* = 3.25e‐10) and STAD (*r* = 0.392, *p* < 2.2e‐16) and monocyte in LIHC (*r* = 0.44, *p* < 2.2e‐16) (Figure S2‐1A). What is more, the *ITGAV* gene expression was the most associated with the infiltration of macrophage in COAD (*r* = 0.466, *p* < 2.2e‐16), natural killer T cell in LIHC (*r* = 0.424, *p* < 2.2e‐16), NK in PAAD (*r* = 0.543, *p* < 2.2e‐16) and STAD (*r* = 0.384, *p* < 2.2e‐16) (Figure S2‐1B). We then analyzed the correlation of *NPC2* and *ITGAV* expression with immune stimulators (Figure 9C,D), immune inhibitors (Figure 9E,F), and major histocompatibility complex (MHC) molecules (Figure 9G,H) in different GICs via the TISIDB database. The Spearman correlations between *NPC2* and *ITGAV* expression with the top four immune stimulators, immune inhibitors, and MHC for each GIC are shown in Supplementary Figure 2‐2.

We also investigated if there was a correlation between immune subtypes and target gene expression. It is important to note that the immune subtypes are divided into six distinct groups: C1 (wound healing), C2 (IFN‐gamma dominant), C3 (inflammatory), C4 (lymphocyte depleted), C5 (immunologically quiet), and C6 (tumor growth factor [TGF‐b] dominant). The results showed that *NPC2* expression levels were significantly related to LIHC and STAD (Figure 9I). The *ITGAV* gene expression was correlated to COAD and STAD immunosubtype (Figure 9J). Finally, we corroborated the immune filtration analysis using data from the TIMER2.0 database. The study's findings indicated that *NPC2* and *ITGAV* expressions positively correlated with CD8+ T cell, CD4+ T cell, B cell, neutrophil, macrophage, and dendritic cell infiltration in nearly all of the chosen GIC tissues (Figure 9K,L). Overall, the data showed that higher levels of immune cell infiltration were connected to overexpression of the genes mentioned above.

### 3.9 Gene Regulatory Network

Transcriptional regulation is a fundamental BP, and considerable effort has been expended worldwide to dissect it and identify its key characters to comprehend how its molecular components work together to regulate gene expression levels [39]. Gene Regulatory Networks (GRNs) are potent instruments for describing and computationally reconstructing the intricate network of interactions underlying the transcriptional regulation of gene expression. GRNs are topological maps that represent and predict molecular entity relationships. On occasion, GRNs consist of regulatory interactions between a TF and its target genes, as well as between noncoding RNAs and target genes as a ceRNA network [40]. The TF and ceRNA regulatory networks as GRNs were the primary focus of our investigation.

#### 3.9.1 NPC2 and ITGAV‐Related TFs Regulatory Network

**FIGURE 10:**
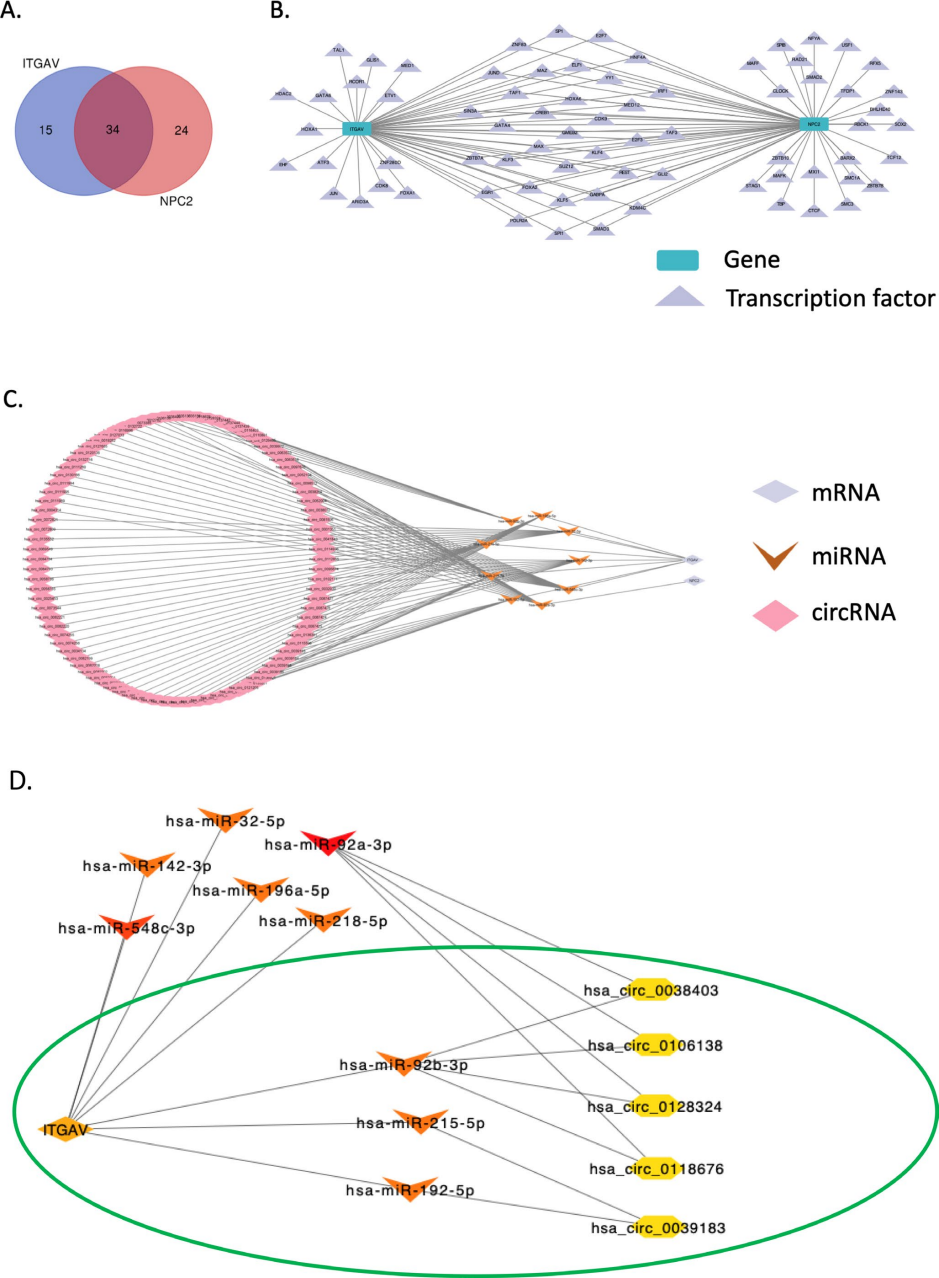
*NPC2* and *ITGAV* regulatory network prediction. (A) Venn diagram of transcription factors of *NPC2* and *ITGAV* based on the prediction of hTFtargets database. (B) TF‐Genes regulatory network prediction. (C) The ceRNA regulatory network of *NPC2* and *ITGAV*. TF: Transcription factor; circRNAs: circular RNAs; miRNAs: micro‐RNAs. (D) The key ceRNA network based on the genes “degree” analyzed by the Cytohubba tool.

TFs are vital components of signal transduction expressed in all human cells. They are generally regarded as the terminal effectors of signaling pathways in cells. As such, the deregulation of a few TFs could profoundly impact overall gene expression profiles. Recent studies have underlined the importance of TFs in cancers [41, 42, 43], and revealed the crucial functions of TFs in tumor initiation, progression, and metastasis [44]. To expound on the role of TFs in the regulation of GICs, we queried the hTFtarget Database for potential TFs for *NPC2* and *ITGAV*, and 58 and 49 TFs were identified, respectively (Table S3). Thirty‐four common candidate TFs were also obtained by the intersection of *ITGAV* and *NPC2* TFs based on hTFtargets through Venn diagram (Figure 10A and Table S4). Eventually, the TF‐Genes regulatory network was visualized with Cytoscape 3.9.1 (Figure 10B).

#### 3.9.2 Prediction of NPC2 and ITGAV mRNA—miRNA—circRNA Regulatory Network

The ceRNA is a novel regulatory mechanism in cancer, which is attracting widespread attention, and emerging evidence demonstrates that the ceRNA hypothesis has played a role in molecular biological mechanisms of occurrence and development of various cancers, including hepatocellular carcinoma, gastric, colorectal, and pancreatic cancer [45, 46, 47, 48]. To better understand the role of circRNAs and miRNAs in the ceRNA network of *NPC2* and *ITGAV* mRNAs, we established a circRNA–miRNA–mRNA (ceRNA) network. As the first step, the miRTarBase tool identified a total of one and eight miRNAs targeting *NPC2* and *ITGAV* mRNA, respectively (Table S5). Further, we retrieved circRNAs data that interact with miRNAs from the CircBank online database and identified a 105 pairs of interacting circRNAs and miRNAs (Table S6). Subsequently, the ceRNA network based on 105 circRNA nodes, 9 miRNA nodes, and 2 mRNA (*NPC2* and *ITGAV*) nodes were constructed and visualized using Cytoscape 3.9.1 (Figure 10C). Ultimately, key regulatory interactions were identified using the Cytohubba tool, based on their “degree” of connectivity. These interactions include ITGAV—hsa‐miR‐92b‐3p–hsa‐circ‐0038403/hsa‐circ‐0106138/hsa‐circ‐0128324/hsa‐circ‐0118676 and ITGAV—hsa‐miR‐215‐5p/hsa‐miR‐192‐5p—hsa‐circ‐0039182 (Figure 10D and Table S7).

### 3.10 Drug–Gene Interaction

The DGIdb was used to identify the drugs interacting with the *NPC2* and *ITGAV*. Table 1 shows the interacted drugs with *ITGAV* genes in addition to related clinical trials in various GICs' cohorts. IMGN‐388, CILENGITIDE, and CYPATE have the highest interaction score for ITGAV (6.47). However, drug interaction for *NPC2* was not indicated by DGIdb.

**TABLE 1:** Drug–gene interactions evaluated via DGIdb and ClinicalTrials.gov databases.

Target gene	Drug	Interaction type	Interaction score	Clinical Trial ID
ITGAV	IMGN‐388	NA	6.37	NA
	CILENGITIDE	Antagonist	6.37	NA
	CYPATE	NA	6.37	NA
	STX‐100	Inhibitor	3.18	NA
	INTETUMUMAB	Antagonist	2.55	NA
	ABITUZUMAB	Inhibitor	2.55	NCT03688230, NCT01008475, and NCT00848510
	VOLOCIXIMAB	NA	2.12	NCT00401570
	ETARACIZUMAB	Antagonist	1.59	NCT00027729 and NCT00284817
	ABCIXIMAB	Inhibitor	1.06	NA
	GLPG‐0187	NA	1.06	NA
NPC2	NA			

## 4 Discussion

Accurately predicting cancer prognosis is of significant value to patients, oncologists, and researchers. Nevertheless, it continues to be a challenge because of the heterogeneity of tumors. With the advent of high‐throughput data, there is a growing expectation that prognostic predictions can be enhanced by integrating clinical and molecular biomarker information. We systematically used several available online databases, pursuing to discover promising biomarkers to predict the prognosis of GIC patients. We first comprehensively analyzed unfavorable prognostic genes among COAD/READ, LIHC, PAAD, and STAD and determined *NPC2* and *ITGAV* as two probable prognostic markers that shared maximum overlap in four GICs. *NPC2* is a protein‐coding gene with a lipid recognition domain that has been demonstrated to be associated with lipoprotein metabolism and innate immune system pathways [32]. High *NPC2* protein expression has been detected in several cancers, such as breast, colon, and thyroid cancer [49, 50]. Emerging pieces of evidence indicate that the *ITGAV* gene leads to the development and progression of various malignancies, such as colon carcinoma, pancreatic adenocarcinoma, esophageal adenocarcinoma, gastric cancer, hepatocellular carcinoma [51, 52, 53, 54, 55, 56]. Multiple mechanisms have been discovered so far to explain how *ITAGV* promotes tumor growth, such as epithelial‐mesenchymal transition (EMT), proliferation, migration, and chemoresistance [53, 57]. However, it was not fully understood how these genes were altered or what role they played in the GICs. Therefore, we used bioinformatics tools to delve further into the functions of these genes in GICs. We conduct an in‐depth investigation of how *NPC2* and *ITGAV* are expressed in human cancers and adjacent normal tissues. Furthermore, we show that *NPC2* expression correlates with LIHC pathological staging, and *ITGAV* expression correlates with LIHC and PAAD pathological staging. In addition, we discovered that in PAAD and COAD, methylation levels of *NPC2* and *ITGAV* are significantly elevated. These results were supplemented by our identification of linkages between *NPC2* and *ITGAV* and the immune signature in the TIME.

In this study, our data suggest that the mRNA expression level of *NPC2* was higher in several tumors, particularly in LIHC, PAAD, and STAD. Besides, *ITGAV* expression was higher in ESCA, PAAD, and STAD. Next, we, on the one hand, found that, in GICs, *NPC2* expression levels were significantly associated with LIHC prognosis (lower OS) and pathological stage. On the other hand, *ITGAV* was correlated with LIHC and PAAD pathological stage significantly but not with other GIC stages. Moreover, we provide evidence that *ITGAV* expression levels were associated with worse OS of LIHC and OS, PFS of STAD patients. These results suggest that *NPC2* and *ITGAV* may be useful as novel pathological staging markers for LIHC, PAAD, and STAD; nevertheless, it would be noteworthy to elucidate further the mechanism by which *NPC2* and *ITGAV* are only linked with LIHC, PAAD, and STAD prognosis or pathological staging.

Cancer refers to a group of illnesses characterized by the unrestrained proliferation of aberrant cells, which is supposed to be predominantly the result of genetic mutations. Some of these mutations are dubbed “drivers” because of their tendency to spur carcinogenesis and provide certain selective advantages for precancerous cells over their neighboring cells. Moreover, it has become apparent that cancer genomes also include several “passenger” mutations. Mutational patterns in driver and passenger genes in cancer genomes shed light on the varied cancer phenotypes [58]. It is interesting to note that our data suggest a mutation rate of 0.5% for *NPC2* and 4% for *ITGAV* in GICs.

Currently, DNA methylation analysis is an emerging method for improving the accuracy of pathological diagnosis and prognosis of several malignancies [59, 60, 61]. We provide preliminary evidence that, according to the UALCAN database, *NPC2* and *ITGAV* DNA methylation levels are reduced in PAAD and COAD, respectively. Based on the UALCAN database, we report preliminary information that promotor methylation levels for *NPC2* and *ITGAV* are increased in PAAD and COAD, respectively. It has been shown that suppressing tumor suppressor genes by hypermethylation of DNA regions in the promoter region may modulate the pathophysiology of GICs. Cancer‐related genes frequently regulate the DNA repair, cell cycle, apoptosis, and tumor‐specific signaling pathways. However, promoter hypermethylation can cause genomic instability [62]. A growing body of research demonstrates a significant relationship between *NPC2* and *ITGAV* genetic aberrations and carcinogenesis. According to earlier research, abnormal *NPC2* expression is significantly linked to a less favorable prognosis for glioblastoma [63]. Using a panel of monoclonal antibodies against *NPC2*, Liao et al. [50] identified *NPC2* overexpression in breast, colon, and lung cancers. In addition, Taguchi et al. [64] confirmed that human and mouse lung adenocarcinoma cells abundantly secreted *NPC2*. It has been demonstrated that *ITGAV* expression increases in a variety of epithelial tumors despite being practically undetectable in normal tissue, which highlights the possible role of integrin expression during cancer development [52]. Integrins participate in cell surface adhesion and signaling and conclusively play crucial roles in cancer development. For illustration, *ITGAV* overexpression stimulated the synergistic effect of integrin and selectin, which promoted adhesion between pancreas cancer cells and peritoneal mesothelial cells, ultimately resulting in the development of PAAD [51]. The association between *ITGAV* expression and poor prognosis in individuals with LIHC was uncovered by Kang et al. [65]. As reported by Kemper et al., high levels of ITGAV expression have been found as a risk factor for PAAD [51]. One point that should be noted is that there are no reports about the association between cancers and methylation of these genes, indicating the novelty of our study. Overall, the fact that *NPC2* and *ITGAV* expression makes it possible to identify numerous cancerous tissues of the GI tract from their noncancerous counterparts highlights the possibility of the aforementioned gene expressions in screening GICs.

Moreover, we carried out a related‐TF prediction for *NPC2* and *ITGAV* and Identified 34 common TFs between these two genes. Most of these TFs have significant roles in cancer development. For instance, the TF Yin Yang 1 (YY1) participates in various biological functions, such as cell proliferation [66], invasion, migration [67], and EMT [68]. Therefore, YY1 is critical for tumor progression, and increasing evidence suggests a close association between YY1 and cancer. Recently, Sato et al. [69] declared that YY1 suppressed the expression of *ITGAV*, and this transcriptional regulation may lead to the suppression of CRC cell migration and invasion. Our findings indicate that the expression of *ITGAV* and *NPC2* may be regulated through ceRNA interactions. The key microRNAs (miRNAs) identified in our network, including hsa‐miR‐92b‐3p, hsa‐miR‐215‐5p, and hsa‐miR‐192‐5p, have been previously implicated in CRC progression [70, 71, 72, 73]. We identified several circRNAs, namely hsa‐circ‐0038403, hsa‐circ‐0106138, hsa‐circ‐0128324, hsa‐circ‐0118676, and hsa‐circ‐0039182 that potentially bind to these miRNAs. This binding could effectively sequester the miRNAs, preventing them from inhibiting their target, ITGAV. However, the specific roles and mechanisms of these circRNAs in cancer progression require further investigation. Future research should focus on experimentally validating these ceRNA interactions and elucidating their functional consequences in GICs development and progression.

We explored the PPI network of *NPC2* and *ITGAV* and found 20 genes associated with *NPC2* and *ITGAV* (10 genes for each). These *NPC2* or *ITGAV*‐related genes all play a role in various cancers. For example, Chen et al. [74] corroborated that low expression of NPC1L1 and NPC2 in HCC tissues might prompt poor clinical outcomes in HCC patients after resection. NPC1L1 and NPC2 combination was an independent prognostic factor for OS and time to recurrence in HCC patients [74]. Furthermore, there is clinical evidence that *ITGB1* expression has been significantly associated with poor prognosis in CRC patients [75]. In conclusion, our discovery of *NPC2*‐ and *ITGAV*‐related genes provides new insights for the diagnosis and treatment of GICs.

Tumor development is profoundly affected by TIME, which consists of tumor cells and infiltrating immune cells. Immune cells were identified as the key component in controlling cancer development among the many TME factors [76, 77]. Therefore, we looked into the relationship between *NPC2* and *ITGAV* and immune cell infiltration in GICs and discovered a positive correlation between these genes and the infiltration of several immune cells, including CD8+ T cells, CD4+ T cells, macrophages, neutrophils, and dendritic cells. CD8+ T cells, known for their cytotoxic capabilities, are crucial in controlling tumor growth and preventing metastasis. Their infiltration into tumors has been linked to favorable prognostic outcomes. Conversely, CD4+ T cells can have dual roles; while they can enhance antitumor immunity, certain subsets may promote tumor progression, particularly in the context of chronic inflammation. The ratio of CD4+ to CD8+ T cells has also been identified as a significant prognostic factor, with higher ratios often correlating with increased metastatic spread and poorer survival rates [77]. Neutrophils are key components of the innate immune response. Their infiltration is often associated with tumor progression due to their roles in promoting inflammation and angiogenesis. Tumors can exploit these immune cells to create a supportive microenvironment that facilitates growth and metastasis [78]. An impressive finding of this study was that the expression of *NPC2* and *ITGAV* were significantly correlated with the abundance of macrophage infiltration in all kinds of aforementioned GICs. According to reports, macrophages upregulate PD‐L1 expression via *TGF‐β*—induced EMT, which plays a crucial role in tumor immunosuppression and immune evasion [79]. Taking into consideration that the expression of *NPC2* and *ITGAV* causes an increase in macrophage infiltration, this finding may provide more evidence for the association between *NPC2* and *ITGAV* expression and a dismal prognosis for GIC patients. In light of the above results, we next evaluated the association between *NPC2* and *ITGAV* with immunomodulators and immunosubtypes. The expression of *NPC2* and *ITGAV* was significantly related to the majority of immune modulators, especially CD274 (PD‐L1), CD96, CSF1R, HAVCR2, and PDCDCDLG2. With the discovery of tumor‐related immune inhibitors, immune checkpoint inhibitors have been extensively used in immunotherapy with remarkable results. Meanwhile, PD‐1/PD‐L1 inhibitors have been authorized as a treatment for various cancers, including COAD, LIHC, PAAD, and STAD [80, 81, 82, 83]. Further, we determined that *ITGAV* expression was linked to COAD and STAD immune subtypes, whereas *NPC2* expression was linked to LIHC and STAD immunosubtypes. The identification of these genes associated with immune cell type infiltration can help in designing targeted therapies that enhance antitumor immune responses. For instance, therapies aimed at increasing CD8+ T cell infiltration or reprogramming macrophages from a pro‐tumor to an antitumor phenotype are currently being explored [77]. Moreover, the use of biomarkers derived from immune cell profiles could improve patient stratification for immunotherapy, ensuring that treatments are tailored to individual immune landscapes.

With the use of the DGIdb database, 10 candidate drugs targeting *ITGAV* were identified. Among them, for Abituzumab (NCT03688230, NCT01008475, and NCT00848510); Volociximab (NCT00401570); and Etaracizumab (NCT00027729 and NCT00284817) clinical trials on GICs' patients have been conducted or are being conducted. In one of these clinical trials, exploratory analyses indicated that abituzumab‐based therapy might be effective in patients with mCRC with high *ITGAV* (particularly αvβ6 member) expression who have otherwise poor prognosis [84].

## 5 Limitations

There are a number of limitations in our study that need further examination. Before anything else, it is important to note that the samples and clinical records in online databases were gathered retrospectively, and as a result, they skewed toward instances with fresh‐frozen specimens of good quality and big tumor sizes in late‐stage patients. Second, there may be uneven prognostic power and overfitting due to the small sample size of some kinds of cancer. Finally, *NPC2* and *ITGAV'*s molecular pathways in cancer are complex and need to be validated in fully independent research before being implemented in clinical practice.

## 6 Conclusion

Taken as a whole, our investigation into *NPC2* and *ITGAV* in GICs revealed detailed information on gene expression, genetic alteration, clinical prognosis, and immune signature. These results will offer a solid basis for future molecular experiments of *NPC2* and *ITGAV* in tumorigenesis and a rationale for developing innovative prognostic and therapeutic approaches for GICs patients.

## Author Contributions

**Moein Piroozkhah:** writing – original draft, writing – review and editing, visualization. **Mohammadreza Zabihi:** conceptualization, investigation, writing – original draft, validation, visualization, formal analysis, methodology. **Pooya Jalali:** writing – original draft, visualization, resources, funding acquisition. **Zahra Salehi:** conceptualization, data curation, supervision, software, project administration.

## Ethics Statement

The authors have nothing to report.

## Consent

The authors have nothing to report.

## Conflicts of Interest

The authors declare no conflicts of interest.

## Supporting information


**FIGURE S1** Co‐expression genes of NPC2 and ITGAV in GICs (LinkedOmics) and pathway enrichment analysis (Enrichr). (A and B) Heat maps show the top 50 significant genes positively or negatively correlated with *NPC2* (A) and *ITGAV* (B) in GICs. (C and D) GEO and KEGG pathway analysis of *NPC2* (C) and *ITGAV* (D) based on the Enrichr database. GICs: Gastrointestinal cancer.
**FIGURE S2‐1**. Correlations between *NPC2* and *ITGAV* Expression and the most infiltrated TIL in COAD, LIHC, PAAD, and STAD based on the TISIDB Database. (A) *NPC2* and (B) *ITGAV*. COAD: Colon adenocarcinoma; LIHC: Liver hepatocellular carcinoma; PAAD: Pancreatic adenocarcinoma; STAD: Stomach adenocarcinoma; GICs: Gastrointestinal Cancers; MHC: major histocompatibility complex.
**FIGURE S2‐2**. Correlations between *NPC2* and *ITGAV* Expression and the expression of Immuno‐regulators in COAD, LIHC, PAAD, and STAD. (A, B) The correlations between the expression of *NPC2* (A) and *ITGAV* (B) and the most common Immuno‐inhibitors among GICs. (C‐F) The correlations between the expression of *NPC2* and *ITGAV* and the top four Immuno‐stimulator (C, D), and MHC molecules (E, F) in each GIC were calculated based on the TISIDB database. COAD: Colon adenocarcinoma; LIHC: Liver hepatocellular carcinoma; PAAD: Pancreatic adenocarcinoma; STAD: Stomach adenocarcinoma; GICs: Gastrointestinal Cancers; MHC: major histocompatibility complex.

**TABLE S1** Unfavorable prognostic genes For COAD/READ, LIHC, PAAD, and SRAD based on the HPA database.
**TABLE S2**. Venn analysis was carried out to investigate the intersection between the unfavorable genes across PAAD, LIHC, STAD, and COAD/READ.
**TABLE S3**. The potential transcription factors for NPC2 and ITGAV based on htfTarget database.
**TABLE S4**. The common candidate TFs were obtained by the intersection of ITGAV and NPC2 transcription factors through Venn Diagram.
**TABLE S5**. Identification of miRNAs targeting NPC2 and ITGAV mRNA, respectively via miRTarBase tool.
**TABLE S6**. Identification of interacted circRNAs with interested miRNAs based on the Circbank database.
**TABLE S7**. Key ceRNA interaction based on degree analyzed with the Cytohubba tool. (Top 15 in network ranked by Degree method).

## Data Availability

The data that support the findings of this study are available in the supplementary material of this article.
