# Supplementary material for: Comprehensive Multi‐Omics Analysis Reveals NPC2 and ITGAV Genes as Potential Prognostic Biomarkers in Gastrointestinal Cancers
Source: Cancer Rep (Hoboken). 2024 Dec 17;7(12):e70087. doi: 10.1002/cnr2.70087 (PMC11652787; doi:10.1002/cnr2.70087)
Supplement: Supplementary file 1 — FIGURE S1 Co‐expression genes of NPC2 and ITGAV in GICs (LinkedOmics) and pathway enrichment analysis (Enrichr). (A and B) Heat maps show the top 50 significant genes positively or negatively correlated with NPC2 (A) and ITGAV (B) in GICs. (C and D) GEO and KEGG pathway analysis of NPC2 (C) and ITGAV (D) based on the Enrichr database. GICs: Gastrointestinal cancer. FIGURE S2‐1. Correlations between NPC2 and ITGAV Expression and the most infiltrated TIL in COAD, LIHC, PAAD, and STAD based on the TISIDB Database. (A) NPC2 and (B) ITGAV. COAD: Colon adenocarcinoma; LIHC: Liver hepatocellular carcinoma; PAAD: Pancreatic adenocarcinoma; STAD: Stomach adenocarcinoma; GICs: Gastrointestinal Cancers; MHC: major histocompatibility complex. FIGURE S2‐2. Correlations between NPC2 and ITGAV Expression and the expression of Immuno‐regulators in COAD, LIHC, PAAD, and STAD. (A, B) The correlations between the expression of NPC2 (A) and ITGAV (B) and the most common Immuno‐inhibitors among GICs. (C‐F) The correlations between the expression of NPC2 and ITGAV and the top four Immuno‐stimulator (C, D), and MHC molecules (E, F) in each GIC were calculated based on the TISIDB database. COAD: Colon adenocarcinoma; LIHC: Liver hepatocellular carcinoma; PAAD: Pancreatic adenocarcinoma; STAD: Stomach adenocarcinoma; GICs: Gastrointestinal Cancers; MHC: major histocompatibility complex. [file CNR2-7-e70087-s001.docx]

**Supplementary data.**

B.

A.

**FIGURE S1 |** **Co-expression genes of NPC2 and ITGAV in GICs (LinkedOmics) and pathway enrichment analysis (Enrichr). (A and B)** Heat maps show the top 50 significant genes positively or negatively correlated with *****NPC2* **(A)** and *ITGAV* **(B)** in GICs. **(C and D)** GEO and KEGG pathway analysis of *NPC2* **(C)** and *ITGAV* **(D)** based on the Enrichr database. GICs: Gastrointestinal cancer.

**FIGURE S2-1 | Correlations between *NPC2* and *ITGAV* Expression and the most infiltrated TIL in COAD, LIHC, PAAD, and STAD** **based on the TISIDB Database**. **(A)** *NPC2* and **(B)** *ITGAV.* COAD: Colon adenocarcinoma; LIHC: Liver hepatocellular carcinoma; PAAD: Pancreatic adenocarcinoma; STAD: Stomach adenocarcinoma; GICs: Gastrointestinal Cancers; MHC: major histocompatibility complex.

**FIGURE S2-2 | Correlations between *NPC2* and *ITGAV* Expression and the expression of Immuno-regulators in COAD, LIHC, PAAD, and STAD.** **(A, B)** The correlations between the expression of *NPC2* **(A)** and *ITGAV* **(B)** and the most common Immuno-inhibitors among GICs. **(C-F)** The correlations between the expression of *NPC2* and *ITGAV* and the top four Immuno-stimulator **(C, D)**, and MHC molecules **(E, F)** in each GIC were calculated based on the TISIDB database. COAD: Colon adenocarcinoma; LIHC: Liver hepatocellular carcinoma; PAAD: Pancreatic adenocarcinoma; STAD: Stomach adenocarcinoma; GICs: Gastrointestinal Cancers; MHC: major histocompatibility complex.
